# Supramolecular Polymer Additives as Repairable Reinforcements for Dynamic Covalent Networks

**DOI:** 10.1002/adma.202410723

**Published:** 2024-10-17

**Authors:** Joost J. B. van der Tol, Shahzad Hafeez, Andy P. G. Bänziger, Hao Su, Johan P. A. Heuts, E. W. Meijer, Ghislaine Vantomme

**Affiliations:** ^1^ Institute for Complex Molecular Systems and Laboratory of Macromolecular and Organic Chemistry Eindhoven University of Technology P.O. Box 513 Eindhoven 5600 MB Netherlands; ^2^ College of Polymer Science and Engineering and State Key Laboratory of Polymer Materials Engineering Sichuan University Chengdu 610065 China

**Keywords:** dynamic covalent network, mechanical properties, reinforcement, repairability, reprocessability, supramolecular polymer additive

## Abstract

Employing rigid (in)organic materials as reinforcement for dynamic covalent networks (DCNs) is an effective approach to develop high‐performance materials. Yet, recycling of these materials after failure often necessitates inefficient chemical reprocessing or inevitably alters their performance due to unrepairable inert components. Here, a non‐covalent reinforcement strategy is presented by introducing a supramolecular additive to a DCN that can reversibly depolymerize and reform on demand, therefore acting as an adaptive and repairable reinforcement. The strong hydrogen‐bonding interactions in the supramolecular polymer of triazine‐1,3,5‐tribenzenecarboxamide (***S***‐**T**) strengthen the DCN at room temperature, while its non‐covalent nature allows for easy one‐pot reprocessing at high temperatures. Depending on wether ***S***‐**T** is covalently bond to the DCN or not, it can play either the role of compatibilizer or filler, providing a synthetic tool to control the relaxation dynamics, reprocessability and mechanical properties. Moreover, the ***S***‐**T** reinforcement can be chemically recovered with high yield and purity, showcasing the recyclability of the composite. This conceptually novel supramolecular reinforcement strategy with temperature‐controlled dynamics highlights the potential of supramolecular polymer additives to replace conventional unrepairable reinforcements.

## Graphical abstract

This study presents an innovative non‐covalent reinforcement strategy for dynamic covalent networks (DCNs) using a supramolecular additive (***S*‐T**) that can depolymerize and reform on demand. Acting as both compatibilizer and filler, ***S*‐T** improves mechanical properties while providing a tool to control processability, thus offering an alternative to traditional non‐repairable reinforcements.

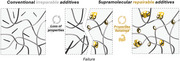

## 1 Introduction

Supramolecular chemistry has evolved as a central science, providing ample opportunities for materials development across various fields such as health^[^ ^1^, ^2^, ^3^, ^4^ ^]^ and energy.^[^ ^5^, ^6^, ^7^, ^8^ ^]^ The intrinsic on‐demand (de)bonding properties of supramolecular materials, arising from the dynamic and labile nature of non‐covalent interactions, have enabled the development of smart, adaptive,^[^ ^9^, ^10^, ^11^ ^]^ and reprocessable materials.^[^ ^12^, ^13^ ^]^ The latter can be unlocked by adjusting the association and dissociation rates, often through heat, which causes the material to relax, flow, and eventually allow malleability.^[^ ^14^, ^15^ ^]^ Here, we aim to extend the function of supramolecular materials as repairable reinforcing additives for polymeric materials.

Currently, dynamic covalent networks (DCNs), also known as covalent adaptable networks (CANs),^[^ ^15^, ^16^, ^17^ ^]^ present a class of materials with great potential to solve the reprocessability and repairability issue of thermosets. However, the use of reinforcing fillers in DCNs,^[^ ^18^, ^19^, ^20^, ^21^, ^22^, ^23^, ^24^ ^]^ similar to those used in industrial thermosets, will not resolve this issue due to a major drawback, namely that these often (in)organic fillers^[^ ^25^ ^]^ break upon failure and remain unrepairable. Recently, the use of carbon nanotubes, fibers, and sheets^[^ ^26^, ^27^ ^]^ as fillers to reinforce DCNs has attracted significant interest due to their ancillary thermal^[^ ^28^ ^]^ and electron‐conductive properties.^[^ ^29^ ^]^ Typically, high contents of (in)organic fillers are required, which often leads to stress focal points, void formation^[^ ^30^ ^]^ and challenges in achieving optimal interfacial compatibility with the polymer matrix.^[^ ^31^, ^32^ ^]^ To release the interfacial stresses between the filler and matrix, block copolymers are often used as compatibilizers, essentially blending into both phases.^[^ ^33^, ^34^ ^]^ Nevertheless, a drawback of these inert fillers is that they alter the mechanical properties of the composite during recycling, as they are inherently unrepairable.^[^ ^35^, ^36^ ^]^ In addition, reprocessing of reinforced DCNs requires labor‐intensive chemical recycling and reimpregnation to restore original properties.^[^ ^37^, ^38^ ^]^ These reports highlight the need for alternative strategies to combine reinforcement and recyclability in DCNs.

Unlike static (in)organic materials, supramolecular polymers are based on non‐covalent intermolecular interactions that facilitate mobility at elevated temperatures before returning to their native state upon cooling.^[^ ^13^, ^39^, ^40^, ^41^ ^]^ This unique feature enables supramolecular polymers to repair themselves after each reprocessing cycle, an approach that could greatly simplify the recyclability of composites by eliminating the need for separation steps. Additionally, these organically composed materials generally exhibit a higher affinity to the polymer matrix compared to purely carbon‐based and inorganic reinforcements, playing the role of thermoreversible organic nanofillers^[^ ^42^ ^]^ and enhancing the final properties of the composite.^[^ ^43^ ^]^ In this context, building blocks capable of forming supramolecular polymers could serve as repairable reinforcements by temperature‐controlled (de)polymerization. For such reinforcement purposes, strong intermolecular interactions are a necessity, as motifs with weak intermolecular interactions will not be able to strengthen the matrix material. We therefore envision that discotic supramolecular monomers, offering strong unidirectional interactions at room temperature and flow‐like properties at processing temperatures, could serve as promising candidates.^[^ ^44^ ^]^ These temperature‐dependent properties have the potential to create versatile and robust composite materials.

**Figure 1:**
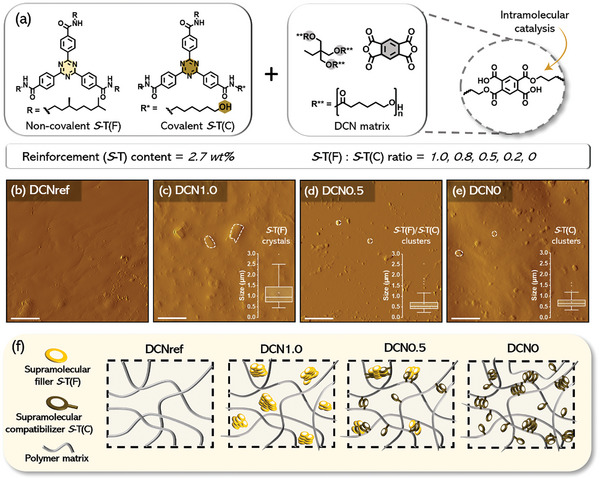
a) Chemical structures of the reinforcements ***S***‐**T(F)** and ***S***‐**T(C)**, the DCN matrix precursors, and the intramolecular catalyzing dynamic covalent motif. A tri‐arm poly(caprolactone) of 2000 g mol^−1^ was used for the synthesis of DCNs. AFM 10 × 10 µm amplitude images of b) **DCNref**, c) **DCN1.0**, d) **DCN0.5** and e) **DCN0** displaying the distinct morphological features between the DCNs as a result of variations in the ***S***‐**T(F)** to ***S***‐**T(C)** ratio. The inset graphs in c),d) and e) display the size distribution of the crystals and/or clusters of ***S***‐**T** for respective DCNs. The inset scale bars represent 2 µm. f) Schematic illustration of the expected change in connectivity, and hence morphology, as the ratio of filler ***S***‐**T(F)** to covalently bonded ***S***‐**T(C)** is varied.

In this work, we present a supramolecular polymer approach that incorporates repairable reinforcements into DCNs to improve their mechanical integrity, while retaining their reprocessability. To establish DCN reinforcement, we use a discotic *C*_3_‐symmetric monomer, triazine‐1,3,5‐tribenzenecarboxamide (***S***‐**T**),^[^ ^45^ ^]^ known for its strong intermolecular interactions relative to other commonly employed *C*_3_‐symmetric monomers, resulting in hydrogen‐bonded supramolecular polymers. We proceed by synthesizing a library of reinforced DCNs in which ***S***‐**T** functions as a compatibilizer, i.e., (***S***‐**T(C)**) covalently bonded to the polycaprolactone‐based DCN through an ester linkage,^[^ ^46^ ^]^ or is simply added as a filler (***S***‐**T(F)**, **Figure** **1**a). By varying the ratio of filler ***S***‐**T(F)** to covalently bonded ***S***‐**T(C)**, we change the microstructure of the reinforcements and are able to fine‐tune both the reprocessability and mechanical properties of the resulting composites. Notably, the reprocessability and mechanical properties could be retained as shown by multiple cycles of tensile testing and compression molding. Finally, we showcase the ability to extract and isolate the ***S*‐T** reinforcements from the DCN matrix using chemical recycling techniques.

## 2 Results and Discussion

### 2.1 Synthesis and Network Characterization

The synthesis of the reinforced DCNs follows a one‐step synthesis comprising the matrix components and either or both the C_3v_‐symmetric ***S***‐**T(F)** and hydroxy‐functionalized ***S***‐**T(C)** (Figure 1a).^[^ ^45^ ^]^ The formation of the DCN matrix involves a reaction between pyromellitic anhydride and a trifunctional hydroxy‐terminated polycaprolactone (PCL) in a 3:2 ratio (Schemes S1 and S2, Supporting Information). This 3:2 ratio ensures that neighboring acid groups remain available for intramolecular catalyzed bond exchange.^[^ ^47^ ^]^ The PCL triol is selected for its well‐defined functionality, as opposed to more industrially relevant (and less defined) branched polyesters, providing full control of the network formation and a comprehensible reference system.^[^ ^46^ ^]^ In order to prevent inhomogeneous mixing, which could potentially deteriorate the mechanical properties, N‐dimethylformamide is used as a solvent, effectively solubilizing both the DCN building blocks and the supramolecular monomers. The complete synthetic description of the supramolecular building blocks ***S***‐**T(C)** and ***S***‐**T(F)**, and the reinforced DCNs is given in Section S2 (Supporting Information).

A library of reinforced DCNs has been synthesized containing a fixed additive content of 2.7 wt.% (2.5 mol%) and variable ratios of covalently bonded ***S***‐**T(C)** and non‐bonded ***S***‐**T(F)** (**Table** **1**). Here, the network **DCN1.0** contains only ***S***‐**T(F)**, **DCN0.8** comprises a mixture of ***S***‐**T(F)** and ***S***‐**T(C)** in a 0.8:0.2 ratio, **DCN0.5** in a 0.5:0.5 ratio, **DCN0.2** a 0.2:0.8 ratio, and **DCN0** solely contains ***S***‐**T(C)**. **DCNref** does not contain any ***S‐*T** additive. All DCNs were characterized using Fourier‐transform infrared (FTIR) spectroscopy (Figure S4, Supporting Information) and the extent of conversion was determined by means of gel content (Table S1, Supporting Information).

**Table 1:** Network characterization and thermal properties of DCNs.

DCN^a)^	*S*‐T content^b)^ [wt.%]	F:C ratioc^)^	Gel fraction^d)^ [%]	*T*_g_ ^e)^ [°C]	*T*_m_ ^f)^ [°C]	*T*_d_ ^g)^ [°C]	*E*_A_ ^h)^ [kJ/mol]
**DCNref**	0	–	97	− 37	–	288	117 ± 6
**DCN1.0**	2.7	1.0	95	− 39	215	311	84
**DCN0.8**	2.7	0.8	91	− 39	213	302	–
**DCN0.5**	2.7	0.5	95	− 42	209	286	116 ± 11
**DCN0.2**	2.7	0.2	92	− 40	192	297	–
**DCN0**	2.7	0.0	92	− 37	211	301	131 ± 6

^a)^ DCNs as depicted in Figure 1;

^b)^ Theoretical weight percentage of ***S***‐**T(C)** and ***S***‐**T(F)** incorporated into the DCN matrix;

^c)^ Weight ratio between ***S***‐**T(F)** and ***S***‐**T(C)** added to the reaction mixture;

^d)^ Gel fraction based on the non‐soluble fraction of synthesized DCNs obtained from a swelling experiment using tetrahydrofuran (THF).

^e)^ Glass transition temperature of the DCN matrix (*T*_g_);

^f)^ Melting temperature of ***S***‐**T(C)** and ***S***‐**T(F)** inside the DCN matrix (*T*_m_);

^g)^ Decomposition temperature of the DCNs based on 5 weight percentage loss (*T*_d_);

^h)^ Activation energy is required to induce dynamic covalent exchange between the ester moieties in the DCNs (*E*_A_).

To study the effect of variations in the additive ratio ***S***‐**T(F)**: ***S***‐**T(C)** on the network connectivity and, consequently, on the morphology, we employed atomic force microscopy (AFM) and polarized optical microscopy (POM). As depicted in Figure 1b, **DCNref** showed a relatively smooth surface devoid of crystalline domains or clusters, indicating the amorphous and homogeneous nature of the matrix. This featureless morphology was corroborated by POM showing no birefringence (Figure S5a, Supporting Information). On the contrary, **DCN1.0** and **DCN0.8** exhibit a granular birefringent POM texture (Figure S5b,c, Supporting Information), illustrating the formation ***S***‐**T** crystals as a result of phase‐segregation driven crystallization. Topological AFM images confirmed this observation, displaying a heterogeneous surface covered with crystallites ranging from 0.5 to 2.5 µm (Figure 1c). This observation differs significantly from **DCN0.5**, **DCN0.2**, and **DCN0**, as no large crystalline structures were observed (Figure S5d–f, Supporting Information). Yet relatively small clusters of ***S*‐T**, ranging from 0.2 to 1.1 µm, were formed as shown by AFM (Figure 1d,e). This implies that the phase segregation between ***S***‐**T** and the matrix occurs even at a low additive content of 2.7 wt.%, but becomes less pronounced, as the content of covalently bonded **S‐T(C)** increases (Figure 1f), and most likely leads to a supramolecular polymer network dominated morphology. These results suggest the function of ***S***‐**T(C)** as a small molecule compatibilizer that is used to promote interfacial adhesion between the polymer matrix and ***S***‐**T(F)**, which have no affinity for each other. The ratio ***S***‐**T(F)**: ***S***‐**T(C)** thus provides a handle to control the microstructure of the material and potentially its mechanical properties and relaxation dynamics. We then attempted to probe the chiral activity of the filled DCN films, as ***S***‐**T(F)** and ***S***‐**T(C)** were expected to form homochiral stacks. However, due to the opacity of the films we were unable to obtain reliable and reproducible results.

### 2.2 Mechanical Properties of Reinforced Dynamic Networks

**Figure 2:**
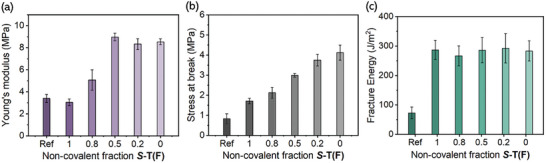
Mechanical properties of the reference and reinforced DCNs (**DCNref**, **DCN1.0**, **DCN0.8**, **DCN0.5**, **DCN0.2**, and **DCN0**) including the a) Young's modulus, b) stress at break, and c) fracture energy. All DCNs are measured in triplicates or quadruplicates.

To gain insight into the influence of the supramolecular additives on the mechanical properties, Young's modulus, stress at break, and fracture energy of DCNs containing 2.7 wt.% (2.5 mol%) of ***S***‐**T** additive were investigated (Figures S6 and S8, Supporting Information). Interestingly, the degree of improvement of Young's modulus strongly depends on the ratio of ***S***‐**T(F)**: ***S***‐**T(C)**, which reaches a maximum at ≈8.5 MPa above an ***S***‐**T(F)**: ***S***‐**T(C)** ratio of 0.5:0.5 (**DCN0.5** → **DCN0**) (**Figure** **2**a). The observed plateau illustrates that further increments in the amount of ***S***‐**T(C)** no longer contribute to the stiffness of the DCN. Notably, at this 0.5:0.5 ratio Young's modulus is more than double the value of the reference material (3.4 MPa vs 8.5 MPa). Conversely, no significant change in Young's modulus was detected by solely incorporating ***S***‐**T(F)** (**DCN1.0**), demonstrating the necessity for a compatibilizer such as ***S***‐**T(C)**. On the contrary, the stress at break is already doubled by the incorporation of the ***S***‐**T(F)** alone (**DCN1.0**), illustrating the reinforcing potential of supramolecular fillers. By increasing the fraction of ***S***‐**T(C)**, the stress at break progressively rises to 4.1 MPa (Figure 2b), quadrupling that of **DCNref** (0.8 MPa), emphasizing the role of ***S*‐T(C)** as a compatibilizer. In addition, the strain at break increased 1.5 times from 27% to 40–45% strain (Figure S6, Supporting Information). This outcome is surprising as this increase in strain goes with an increase in stress at break, which is counterintuitive given the typically observed inverse relationship between tensile strength and ductility.^[^ ^48^, ^49^ ^]^

We then explored the ability of the supramolecular additives to resist fracture propagation and dissipate the released energy using a single‐edge notch test. Remarkably, the fracture energy quadrupled with respect to **DCNref** to a value of ≈280 J m^−2^ for all reinforced DCNs (Figure 2c). The fracture energy remained nearly unaffected by the ***S***‐**T(F)**: ***S***‐**T(C)** ratio, implying that the ability of the DCNs to resist fracturing (toughness) is nearly independent of the microstructure of the ***S***‐**T** aggregates. In this context, the current strategy thus allows toughness improvement without the need for chemical integration of the reinforcement additive into the DCN matrix. In addition, we observed that the impact of the supramolecular additive content (2.7 vs 5 wt.%) was negligible or slightly detrimental for the Young's modulus (5 vs 4.5 MPa) and stress at break (2.1 vs 1.5 MPa; Figure S7, Supporting Information) for the highest content. These findings highlight that only a small fraction of supramolecular additive is sufficient to effectively reinforce the DCN, opposed to conventional reinforcement strategies that often require >10 wt.% of additive.^[^ ^30^, ^50^ ^]^

### 2.3 Thermal Properties and Dynamics of Reinforced DCNs

**Figure 3:**
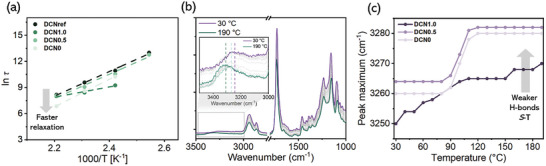
a) Arrhenius plots displaying the variation in relaxation times of the reinforced DCNs with respect to **DCNref**. b) FTIR spectra of **DCN0.5** as a function of temperature. Inset figure: the shift of the N─H_stretch_ vibration to higher wavenumbers (purple to blue dotted lines) and the appearance of the vibration at 3348 cm^−1^ (green dotted line), which is indicative of the weakening and eventual loss of hydrogen bonds (H─bonds) between the ***S***‐**T** additives. c) The corresponding shift in cm^−1^ of the N─H_stretch_ vibration as a function of temperature for **DCN1.0**, **DCN0.5**, and **DCN0**.

Sparked by the improved mechanical properties, we aimed to elucidate the molecular origin of the reinforcement by studying the rearrangement dynamics of both the network and the ***S***‐**T** additive. Accordingly, stress relaxation experiments were performed on **DCN1.0**, **DCN0.5**, **DCN0**, and **DCNref** at various temperatures (120, 140, 160 and 180 °C, Figure S10, Supporting Information). Subsequently, their activation energies for network rearrangement (*E*_A_) could be obtained from these curves (Table 1). As shown in **Figure** **3**a, **DCN0** and **DCN0.5**, show a stronger temperature dependence than **DCN1.0**. As both these DCNs comprise covalently bonded ***S***‐**T(C)**, the strong temperature dependence is most likely a contribution of additional non‐covalent crosslinks. The distinct temperature‐dependent profile of **DCN1.0** and its faster relaxation at lower temperatures is attributed to a lower crosslink density caused by phase‐separated ***S***‐**T(F)** crystals that locally impede network formation. Interestingly, **DCN0.5** and **DCN0** display faster reorganization dynamics than **DCNref**, which becomes increasingly apparent at higher temperatures. This mobility might be associated with the dissociation of non‐covalent interactions between ***S***‐**T** monomers at elevated temperatures, although differential scanning calorimetry (DSC) only indicated melting temperatures (*T*_m_) above 200 °C (Figure S12 and Table 1, Supporting Information). To rule out the possibility of degradation, thermogravimetric analysis (TGA) was carried out, which, gratifyingly, did not reveal any considerable degradation below 280 °C for all DCNs (Figure S13 and Table 1, Supporting Information).

To gain a better understanding of the effect of the intermolecular hydrogen bonding strength between the ***S***‐**T** cores on the DCN dynamics, we monitored the changes in the vibrational spectra of **DCN0.5** as a function of temperature (Figure 3b,c). Upon heating, a clear shift in the N─H stretching vibration was observed from 3264 to 3282 cm^−1^ over a temperature range of 80–120 °C. This shift suggests a weakening of the hydrogen bonding interaction between ***S***‐**T** monomers, as the DCN matrix typically starts to reorganize ≈100 °C.^[^ ^46^ ^]^ In addition, a shoulder appears at 3348 cm^−1^ from 130 °C onwards, indicating further attenuation or even disruption of the hydrogen bonds (Figures 3b; S16, Supporting Information). Similar spectral changes were observed for **DCN0**, but for **DCN1.0**, the shift in the N─H_stretch_ peak appeared to be more gradual, starting at a lower wavenumber (3250 cm^−1^), and therefore indicating stronger hydrogen bonding (Figures 3c and S14–S18, Supporting Information). This variance is likely caused by the higher fraction of ***S***‐**T(F)** monomers present as crystalline aggregates in **DCN1.0**. Consequently, the mobility of ***S***‐**T** is hampered and less reliant on the dynamics of the DCN matrix. This observation is in accordance with the distinct temperature‐dependent profile of **DCN1.0** as demonstrated in Figure 3a. Importantly, these results demonstrate that the mobility of ***S***‐**T** within the DCNs increases prior to the respective melting temperatures and thus contributes significantly to the overall network relaxation.

### 2.4 Repairability and Recoverability of Supramolecular Reinforcements

**Figure 4:**
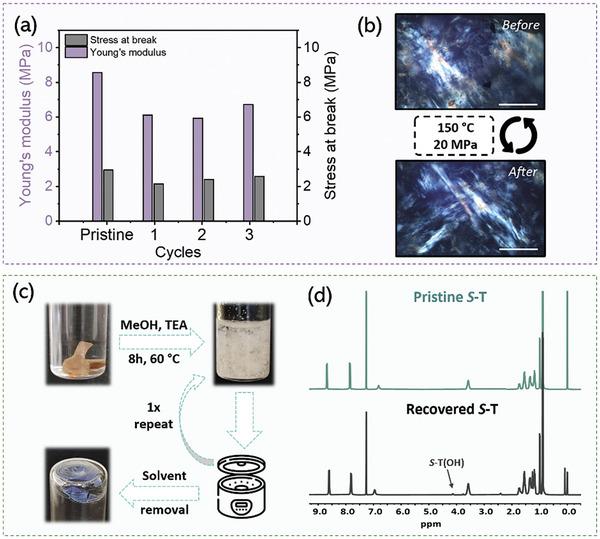
a) Bar diagram displaying the retention of mechanical properties for **DCN0.5** over multiple reprocessing cycles with respect to Young's modulus and stress at break. b) POM images showing similar birefringent structures before and after reprocessing of **DCN0.5**, thereby demonstrating the repairability of the supramolecular additive ***S***‐**T**. The images are taken at 60 °C to exclude birefringence from crystalline polycaprolactone regions. The inset scale bar represents 0.5 mm. c) Photographs illustrating the ability to recover and isolate the reinforcement additives ***S***‐**T(F)** and ***S*‐T(C)** by chemical recycling. d) ^1^H NMR spectra of pristine ***S***‐**T(F)** (top) and recovered ***S***‐**T(F)** and ***S***‐**T(C)** (bottom) in CDCl_3_/TFA*_d1_*.

A key attribute of DCNs is that their properties can be retained after multiple cycles of recycling, which, in the case of reinforced DCNs, strongly depends on the repairability of the reinforcing additive. To this end, the mechanical integrities of **DCNref** and **DCN0.5** were assessed through a series of tensile tests involving various cycles of cutting and compression molding (Figure S19, Supporting Information). As shown in **Figure** **4**a, Young's modulus of **DCN0.5** decreases slightly in the first cycle from 8.5 to 6.1 MPa, and then remains stable until the third cycle. On the contrary, the stress at break shows minimal variation and remains constant at 2.4 MPa, although slightly lower than the pristine material (2.9 MPa; Figure 4a). These results demonstrate the effective retention of the mechanical properties of **DCN0.5** and, more importantly, the repairability of reinforcement ***S***‐**T**. The repairability of ***S*‐T** was further confirmed by the fact that after reprocessing, ***S*‐T** regained a similar birefringent structure as shown by POM. (Figure 4b). A similar outcome was observed for **DCNref** (Figure S19b, Supporting Information), demonstrating the retention of mechanical properties for both filled and unfilled DCNs over multiple cycles. These observations were in line with rheological measurements using four consecutive stress relaxation experiments at 160 °C, revealing no substantial alterations (Figure S11, Supporting Information).

Another attractive feature that arises from the ability of supramolecular reinforcements to reversibly assemble/disassemble is their recoverability and subsequent reuse. This prompted us to demonstrate the potential of isolating the two reinforcement additives, ***S***‐**T(C)** and ***S***‐**T(F)**, from the DCN precursors by chemical recycling. We thereby selected **DCN0.5** as it contains both elements. We started by immersing 0.2 g of **DCN0.5** in 2 mL of methanol containing triethylamine (100 µL) as a catalyst, for 8 h at 60 °C. In this process, methanol induces both the degradation of the DCN network through transesterification and acts as a bad solvent, leading to the precipitation of ***S***‐**T(F)** and ***S***‐**T(C)**. Remarkably, the separation of the white precipitate using centrifugal forces followed by a second cycle of transesterification and precipitation resulted in a recovery of ***S***‐**T** molecules in 63% yield with a purity exceeding 95% (Figures 4c,d; S20, Supporting Information). This approach effectively demonstrates the ability to recover the supramolecular additives from the DCN matrix. A detailed description of the experiments is given in section S8 (Supporting Information).

## 3 Conclusion

This study presents an alternative reinforcement strategy using supramolecular additives that reversibly depolymerize and reform after reprocessing, showcasing the great potential to replace conventional unrepairable reinforcements. The supramolecular polymer additive ***S***‐**T** significantly improves the mechanical properties of DCNs at room temperature while their desirable reprocessability and repairability are preserved. Where supramolecular building blocks with weak intermolecular interactions fail to significantly reinforce DCNs, the addition of only 2.7 wt.% of the supramolecular monomer ***S***‐**T** with strong unidirectional interactions, acting as a filler, quadrupled the DCN toughness. This achievement stands in contrast to related reinforcement strategies using much higher contents of additives. By incorporating covalently bonded ***S***‐**T**, which essentially acts as a small molecule compatibilizer between the supramolecular additive and DCN, we were able to control the microstructure and mechanical properties of the material.

In contrast to the often labor‐intensive chemical recycling of reinforced DCNs using inert additives, the non‐covalent nature of the supramolecular additives allows for easy one‐pot reprocessing. In addition, their incorporation provides control of the network relaxation times without compromising the repairability of the reinforced DCNs, unlike what is often observed with inorganic reinforcements. Importantly, the ability to recover the supramolecular reinforcements further differentiates our work from conventional strategies where the reinforcements are often irreversibly bonded to the matrix material to enhance interfacial compatibility. While this work serves as an important first step toward repairable fiber‐reinforced DCNs, future work will be required to improve their mechanical properties.^[^ ^51^, ^52^ ^]^ We anticipate that this conceptually novel approach will open up new avenues for the design and fabrication of repairable additives for composite materials.

## Conflict of Interest

The authors declare no conflict of interest.

## Author Contributions

The manuscript was written through the contributions of all authors. All authors have given approval to the final version of the manuscript.

## Supporting information



Supporting Information

## Data Availability

The data that support the findings of this study are available in the supplementary material of this article.
