# Supplementary material for: Supramolecular Polymer Additives as Repairable Reinforcements for Dynamic Covalent Networks
Source: Adv Mater. 2024 Oct 17;36(49):2410723. doi: 10.1002/adma.202410723 (PMC11619224; doi:10.1002/adma.202410723)
Supplement: Supplementary file 1 — Supporting Information [file ADMA-36-2410723-s001.pdf]

ADVANCED MATERIALS

Supporting Information

for *Adv. Mater.*, DOI 10.1002/adma.202410723

Supramolecular Polymer Additives as Repairable Reinforcements for Dynamic Covalent Networks

*Joost J. B. van der Tol, Shahzad Hafeez, Andy P. G. Bänziger, Hao Su, Johan P. A. Heuts, E. W. Meijer and Ghislaine Vantomme**

Supramolecular polymer additives as repairable reinforcements for dynamic covalent networks

Joost J. B. van der Tol,[a] Shahzad Hafeez,[a] Andy P. G. Bänziger,[a] Hao Su,[a,b] Johan P. A. Heuts,[a] E. W. Meijer,[a] Ghislaine Vantomme*[a]

[a] Dr. J.J.B.van der Tol, Dr. S. Hafeez, A. P. G. Bänziger, Dr. J.P.A Heuts, Prof. Dr. E.W. Meijer and Dr. G. Vantomme*.
Institute for Complex Molecular Systems and Laboratory of Macromolecular and Organic Chemistry, Eindhoven University of Technology, P.O. Box 513, 5600 MB Eindhoven (The Netherlands)

[b] Dr. H. Su
College of Polymer Science and Engineering and State Key Laboratory of Polymer Materials Engineering, Sichuan University, Chengdu 610065 (China)

Table of Contents

1. Materials and Methods 2
2. Synthesis of supramolecular reinforcements and dynamic covalent networks 3
   2.1 Synthesis of supramolecular reinforcements ***S*-T(F)** and ***S*-T(C)** 3
   2.2 Synthesis of dynamic covalent networks. 4
   2.3 NMR spectra of ***S*-T(F)** and ***S*-T(C)** 6
3. Material characterization of dynamic covalent networks 7
   3.1 Gel fraction of dynamic covalent networks. 7
   3.2 FTIR spectra of dynamic covalent networks. 8
   3.3 POM images of dynamic covalent networks. 8
4. Mechanical properties of dynamic covalent networks 9
   4.1 Tensile experiments. 9
   4.2 Single-edge notch experiments in tensile mode. 10
5. Stress relaxation experiments 11
6. Thermal properties of dynamic covalent networks 13
7. Variable temperature infrared spectroscopy 14
8. Recycling & recovery experiments 15
   8.1 Recycling and reinforcement recovery procedure. 15
   8.2 Tensile experiments on recycled **DCNref** and **DCN0.5**. 16
   8.3 ^{1}H NMR spectra of ***S*-T** upon chemical recycling of **DCN0.5**. 16
9. References 17

1. Materials and Methods

All reagents were purchased from commercial resources and used without further purification. Solvents were purchased from Biosolve and dry solvents were obtained using the MBraun solvent purification system (MB SPS-800). Oven-dried glassware (120 °C) was used for all reactions carried out under argon atmosphere. Deuterated compounds were obtained from Cambridge Isotopes Laboratories.

NMR spectra were recorded using a Varian Mercury Vx 400 MHz (^{1}H NMR using 400 MHz and ^{13}C NMR using 100 MHz). Proton and carbon chemical shifts are reported in ppm (δ) downfield from tetramethylsilane (TMS) using the deuterated solvent resonance frequency as internal standard. Peak multiplicities are abbreviated as s: singlet; d: doublet; t: triplet; q: quartet; p: pentet; m: multiplet; dd: double doublet; dt: double triplet and dq: double quartet.

A Heraeus Megafuge 1.0 Centrifuge was used to separate precipitates from the solvent.

Matrix assisted laser absorption/ionization mass time of flight (MALDI-TOF) measurements were performed on a Bruker Autoflex Speed using α-cyano-4-hydroxycinnamic acid (CHCA) and *trans*-2-[3-(4-*tert*-butylphenyl)-2-methyl-2-propenylidene]malononitrile (DCBT) as matrices.

Variable temperature infrared spectroscopy (VT-IR) was conducted using a Bruker Tensor 27 PMA50 FT-IR spectrometer equipped with a PIKE hot stage. The samples were subjected to a heating and cooling run from 30 °C to 190 °C with a rate of 5 K min^{-1}.

Polarized optical microscopy (POM) images were taken by a Jenaval polarization microscope with crossed polarizers. The images were obtained from the non-processed materials using a 4x magnification.

Differential scanning calorimetry (DSC) was performed on a DSC Q2000 from TA instruments, calibrated with an indium standard. The samples (6−10 mg) were weighed directly into aluminium pans and hermetically sealed. The samples were first heated to 250 °C followed by two cooling/heating cycles from −70 °C to 250 °C with a rate of 10 K min^{-1}. The represented data displays the second heating and cooling run. All samples were dried at 120 °C at 21 mbar for 16 hours in a vacuum oven prior to the measurement.

Thermogravimetric analysis (TGA) was realized using a TGA550 from TA Instruments. The sample was first heated to 100 °C for 30 minutes followed by a heating run from 100 °C to 800 °C at 10 K min^{-1} while recording the sample's weight. All samples were dried at 120 °C at 21 mbar for 16 hours in a vacuum oven prior to the measurement.

Tensile experiments were carried out on a DMA Q850 (TA Instruments) using a film tension set-up. For all measurements, a constant strain rate of 5 mm/min, a preload force of 0.1 N and a force track of 125% were used. The stress was recorded as a function of strain. Samples were prepared *via* compression moulding (25 [length] x 10 [width] x 0.7 [thickness] mm) at 150 °C and 20 MPa for 2 hours. In a next step, the reshaped samples were slowly cooled to room temperature before removal followed by cutting them into the desired dogbone shape (25 [length] x 2 [width] x 0.7 [thickness] mm). Prior to each measurement, the dogbone samples were dried at 120 °C at 21 mbar for 16 hours in a vacuum oven.

Rheological measurements were performed on a strain-controlled AR-G2 rheometer with an ETC oven setup (TA Instruments) and parallel-plate geometry. For all experiments, a constant force of 1 N was applied in order to ensure proper contact between the plates and the sample. Prior to each measurement, the samples were thermally treated at 180 °C for 5 hours to eliminate potential measurement inconsistencies regarding different degrees of curing or sample roughness. Stress relaxation data were acquired using a step strain of 1% (within the viscoelastic regime) and the relaxation modulus G(t) monitored over time at various temperatures. Samples were prepared *via* compression moulding (8 [diameter] x 0.7 [thickness] mm) at 150 °C and 20 MPa for 2 hours. All samples were dried at 120 °C at 21 mbar for 16 hours in a vacuum oven prior to the measurement.

2. Synthesis of supramolecular reinforcements and dynamic covalent networks

*2.1 Synthesis of supramolecular reinforcements **S**-***T(F)*** and **S**-***T(C)***

*Synthesis of 4,4',4''-(1,3,5-triazine-2,4,6-triyl)tris(N-((S)-3,7-dimethyloctyl) benzamide) [**S**-**T**(C)]*

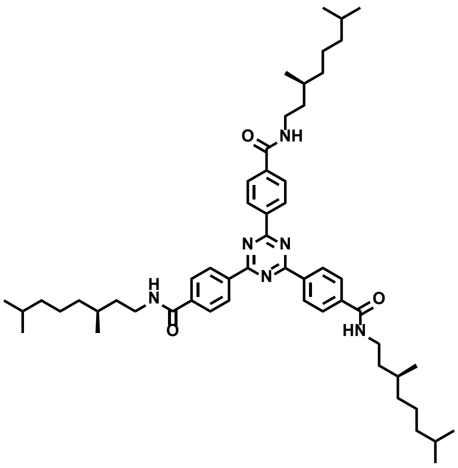

A previously synthesized batch of chiral *S*-triazine (***S*-T**), from which the synthesis has been published before,[1] was used for all experiments. ^{1}H NMR (400 MHz, Chloroform-d_1 and 3 vol% TFA- d_1): δ [ppm] = 8.57 (s, 6H), 7.79 (s, 6H), 3.57 (s, 6H), 1.74 (s, 3H), 1.64 – 1.48 (m, 9H), 1.41 – 1.11 (m, 18H), 0.99 (d, ^3J = 5.6 Hz, 9H), 0.89 (d, ^3J = 6.5 Hz, 18H). ^{13}C NMR (101 MHz, Chloroform-d_1 and 3 vol% TFA-d_1): δ [ppm] = 170.70, 169.80, 138.95, 136.49, 129.54, 127.53, 39.65, 39.34, 37.22, 36.28, 31.06, 28.12, 24.81, 22.78, 22.68, 19.50. MALDI-TOF-MS found: 859.31 m/z (calculated [M+H]$^+$: 859,61). FT-IR (cm^{-1}): 3256, 2956, 2923, 2870, 1631, 1579, 1545, 1517, 1467, 1406, 1370, 1313, 1259, 1145, 1099, 1018, 869, 821, 742, 698, 677, 631, 532.

*Synthesis of 4,4'-(6-(4-((6-hydroxyhexyl)carbamoyl)phenyl)-1,3,5-triazine-2,4-diyl)bis(N-((S)-3,7-dimethyloctyl)benzamide) [**S**-**T**(C)]*

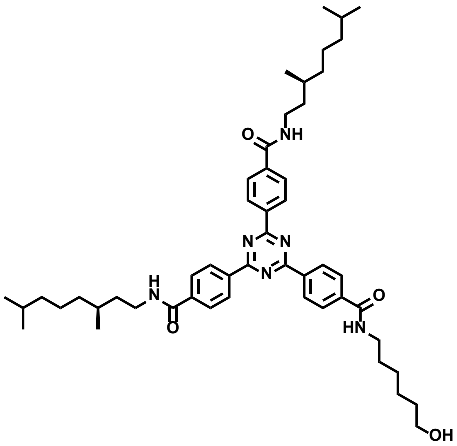

2 g of 4,4',4''-(1,3,5-triazine-2,4,6-triyl)tribenzoic acid (4.5 mmol) was suspended in 40 mL DMF in a 100 mL round-bottom flask. Subsequently, HATU (5.2 g, 13.9 mmol, 3.1 eq.) and DIPEA (2.11 g, 16.4 mmol, 3.6 eq.) were added and the mixture was stirred for 20 min. After that, 1.57 g of (S)3,7-dimethyloctan-1-amine (10.0 mmol, 2.2 eq.) and 0.59 g of 6-amino-1-hexanol (5 mmol, 1.1 eq.) were dropwise added into the mixture and the reaction was carried out at 50 °C for 2 h. The solution was cooled down and diluted with 250 mL chloroform, and washed with 1M HCl (250 mL), brine (250 mL) and water (250 mL). The organic layer was collected, dried over MgSO4, and concentrated via rotary

evaporation. The crude product was first purified by column chromatography using $CHCl_3$/EtOAc (gradient of 100/0 to 40/60) as eluent followed by a second column using $CHCl_3$/MeOH (gradient of 90/10 to 85/15) as eluent. Then the solution was concentrated and precipitated in cold methanol. The pure product was obtained via filtration and dried as a solid with a metallic appearance (yield 16%, 590 mg, 0.72 mmol).^{1}H NMR (400 MHz, Chloroform-d_1 and 3 vol% TFA- d_1): δ [ppm] = 8.58 (d, ^{3}J = 8.0 Hz, 6H), 7.80 (d, ^{3}J = 8.1 Hz, 6H), 7.08 (s, Hz, 3H), 3.86 (t, ^{3}J = 6.5 Hz, 2H), 3.58 (m, 6H), 1.69 – 1.14 (m, 28H), 0.99 (d, ^{3}J = 6.3 Hz, 6H), 0.89 (d, ^{3}J = 6.6 Hz, 12H). ^{13}C NMR (101 MHz, Chloroform-d_1 and 3 vol% TFA-d_1): δ [ppm] = 170.66, 170.03, 161.76, 161.32, 160.89, 160.46, 129.45, 127.43, 118.53, 115.70, 112.87, 110.04, 68.21, 39.72, 39.22, 37.08, 36.11, 30.92, 28.95, 28.00, 26.35, 25.22, 24.68, 22.66, 22.56, 19.37, 18.57. MALDI-TOF-MS found: 819.25 m/z (calculated $[M+H]^+$: 819.55). FT-IR (cm^{-1}): 3252, 2956, 2926, 2868, 1786, 1632, 1580, 1545, 1517, 1466, 1407, 1371, 1314, 1219, 1146, 1018, 870, 821, 730, 699, 678, 630, 532.

2.2 Synthesis of dynamic covalent networks.

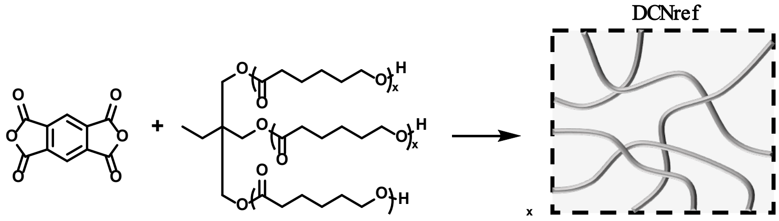

Scheme S1. Synthesis of dynamic covalent network **DCNref**. Reaction conditions*:* dry DMF i) 100 °C, 24 h ii) 150 °C, 48 h.

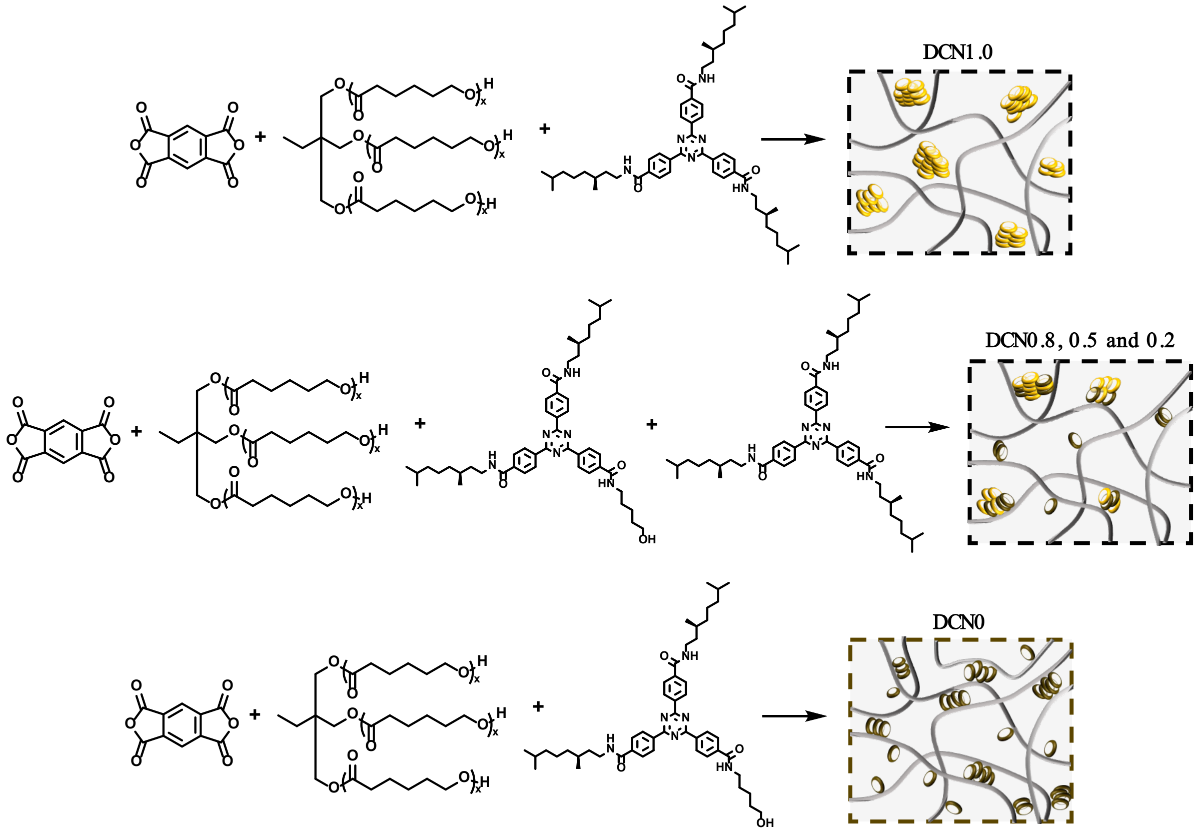

Scheme S2. Synthesis of dynamic covalent networks **DCN1.0**, **DCN0.8**, **DCN0.5**, **DCN0.2** and **DCN0**. Reaction conditions: dry DMF i) 100 °C, 24 h ii) 150 °C, 48 h.

Synthesis of DCNref.

Pyromellitic anhydride (PMDA) (1 g, 4.6 mmol) in 6 mL dry DMF was transferred into a 50 mL flask. Subsequently, a tri-arm polycaprolactone (5.9 g, 3 mmol) in 10 mL dry DMF was added to the flask and heated to 100 °C. After refluxing for 24 hours, the reflux condenser was removed and the temperature increased to ~150 °C to slowly evaporate the DMF from the reaction mixture. After DMF was evaporated, the formed network was further cured and vacuum dried in the oven at 130 °C for 48 h yielding the product as a transparent to slightly yellow polymer network.[2]

General procedure for the synthesis of reinforced DCNs.

PMDA (0.5 g, 2.3 mmol) in 3 mL dry DMF was transferred into a 50 mL round-bottom flask. Subsequently, ***S*-T(F)** and ***S*-T(C)** (0.098 mmol) in 6 mL dry DMF were added to the flask in varying molar ratios (1:0, 1:4, 1:1, 4:1 and 0:1) followed by the addition of a tri-arm polycaprolactone (2.95 g, 1.5 mmol) in 5 mL dry DMF. Subsequently, the reaction mixture was heated to 100 °C for 24 hours, after which the reflux condenser was removed and the temperature increased to ~150 °C to slowly evaporate the DMF from the reaction mixture. After DMF was evaporated, the formed network was further cured and vacuum dried in the oven at 130 °C for 48 h yielding the product as a slightly yellow and transparent to opaque polymer network.

DCN1.0: Starting from PMDA (0.5 g, 2.3 mmol), ***S*-T(F)** (0.084 g, 0.098 mmol) and tri-arm polycaprolactone (2.95 g, 1.5 mmol), **DCN1.0** could be obtained using the general procedure for the synthesis of reinforced DCNs as described above.

DCN0.8: Starting from PMDA (0.5 g, 2.3 mmol), ***S*-T(F)** (0.065 g, 0.075 mmol), ***S*-T(C)** (0.018 g, 0.023 mmol) and tri-arm polycaprolactone (2.95 g, 1.5 mmol), **DCN1.0** could be obtained using the general procedure for the synthesis of reinforced DCNs as described above.

DCN0.5: Starting from PMDA (0.5 g, 2.3 mmol), ***S*-T(F)** (0.039 g, 0.049 mmol), ***S*-T(C)** (0.042 g, 0.049 mmol) and tri-arm polycaprolactone (2.95 g, 1.5 mmol), **DCN1.0** could be obtained using the general procedure for the synthesis of reinforced DCNs as described above.

DCN0.2: Starting from PMDA (0.5 g, 2.3 mmol), ***S*-T(F)** (0.016 g, 0.19 mmol), ***S*-T(C)** (0.64 g, 0.079 mmol) and tri-arm polycaprolactone (2.95 g, 1.5 mmol), **DCN1.0** could be obtained using the general procedure for the synthesis of reinforced DCNs as described above.

DCN0: Starting from PMDA (0.5 g, 2.3 mmol), ***S*-T(C)** (0.079 g, 0.098 mmol) and tri-arm polycaprolactone (2.95 g, 1.5 mmol), **DCN1.0** could be obtained using the general procedure for the synthesis of reinforced DCNs as described above.

DCN(5)0.8: Starting from PMDA (0.5 g, 2.3 mmol), ***S*-T(F)** (0.129 g, 0.15 mmol), ***S*-T(C)** (0.036 g, 0.045 mmol) and tri-arm polycaprolactone (2.95 g, 1.5 mmol), **DCN(5)0.8** could be obtained using the general procedure for the synthesis of reinforced DCNs as described above.

*2.3 NMR spectra of **S**-***T(F)*** and **S**-**T(C)***

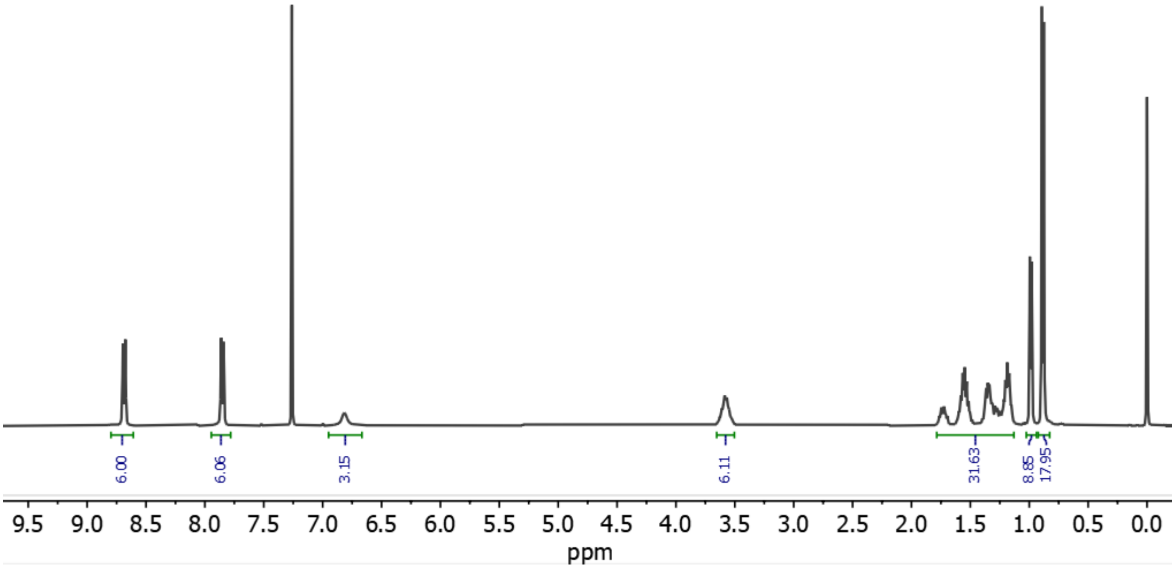

Figure S1. ^{1}H NMR spectrum of pristine ***S*-T(F)** in $CDCl_3$/TFA_{d1} (400 MHz, 25 °C).

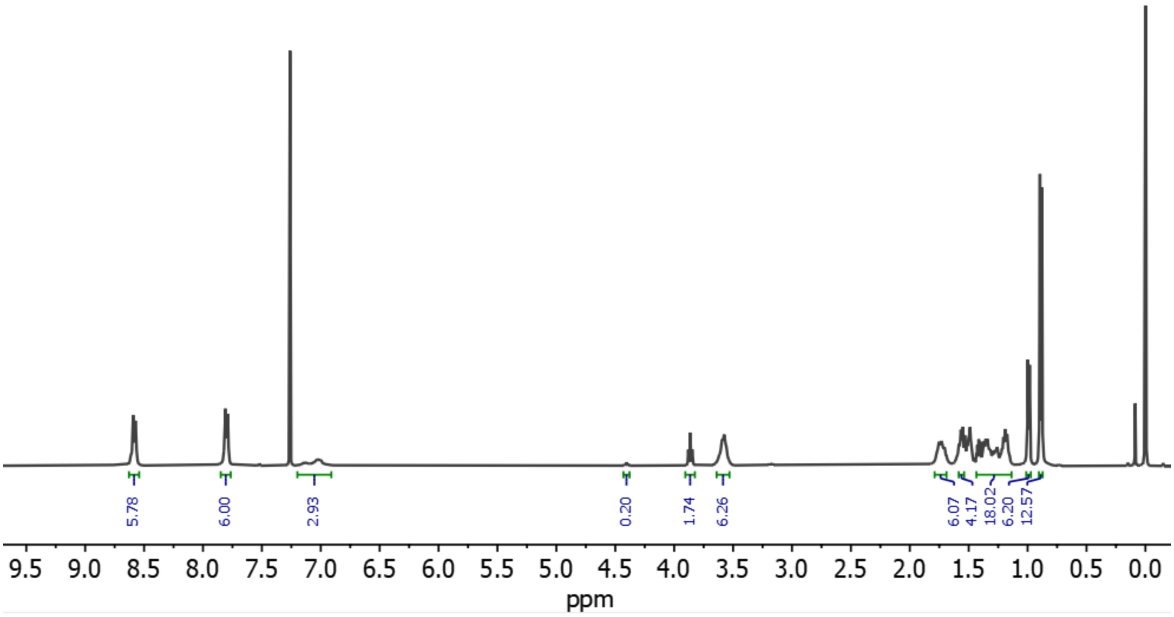

Figure S2. ^{1}H NMR spectrum of pristine ***S*-T(C)** in $CDCl_3$/TFA_{d1} (400 MHz, 25 °C).

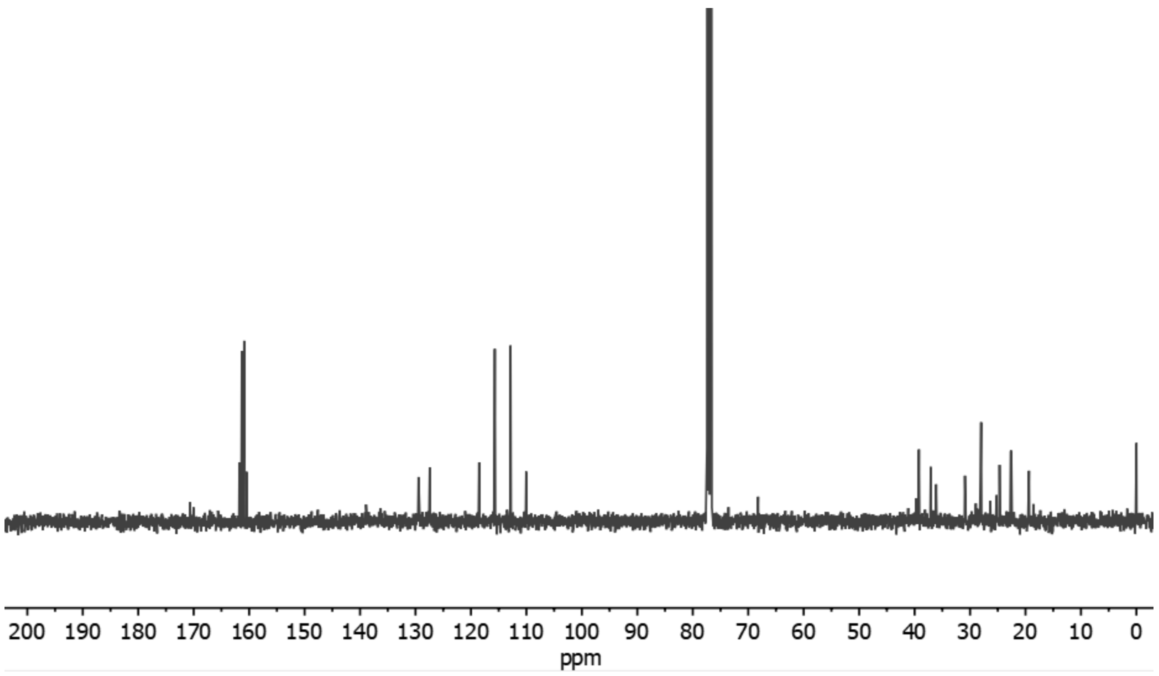

Figure S3. ^{13}C NMR spectrum of pristine ***S*-T(C)** in $CDCl_3/TFA_{d1}$ (100 MHz, 25 °C).

3. Material characterization of dynamic covalent networks

3.1 Gel fraction of dynamic covalent networks.

The gel fraction was determined by the ratio of the mass before and after swelling in THF (Eq. S1). First, 0.05 g material was dried at 120 °C for 2 days ($m_{initial}$) followed by swelling the material in 2 mL of THF for 1 hour. Subsequently, the solvent was removed and replaced by fresh THF. The swelling and solvent removal was repeated at least three times before the material was dried at 100 °C for 2 days (m_{dry}).

$$gel\ fraction\ (\%) = \frac{m_{dry}}{m_{initial}} \times 100\% \qquad \text{(S1)}$$

Table S1. Gel fraction of synthesized dynamic covalent networks.

DCN[a]	***S*-T** content[b] [mol%]	***S*-T** content[c] [wt%]	NC:C ratio[d]	Gel fraction[e] (%)
**DCNref**	0	0	-	97
**DCN1.0**	2.5	2.7	1.0	95
**DCN0.8**	2.5	2.7	0.8	91
**DCN0.5**	2.5	2.7	0.5	95
**DCN0.2**	2.5	2.7	0.2	92
**DCN0**	2.5	2.7	0.0	92
**DCN(5)0.8**	5	5.4	0.8	92

[a] DCNs as depicted in Figure 1. [b] Theoretical molar percentage of ***S*-T(C)** and ***S*-T(F)** incorporated into the DCN matrix. [c] Theoretical weight percentage of ***S*-T(C)** and ***S*-T(F)** incorporated into the DCN matrix. [d] Weight ratio between ***S*-T(C)** and ***S*-T(F)** added to the reaction mixture. [e] Gel fraction based on the non-soluble fraction of synthesized DCNs obtained from a swelling experiment using tetrahydrofuran (THF).

3.2 FTIR spectra of dynamic covalent networks.

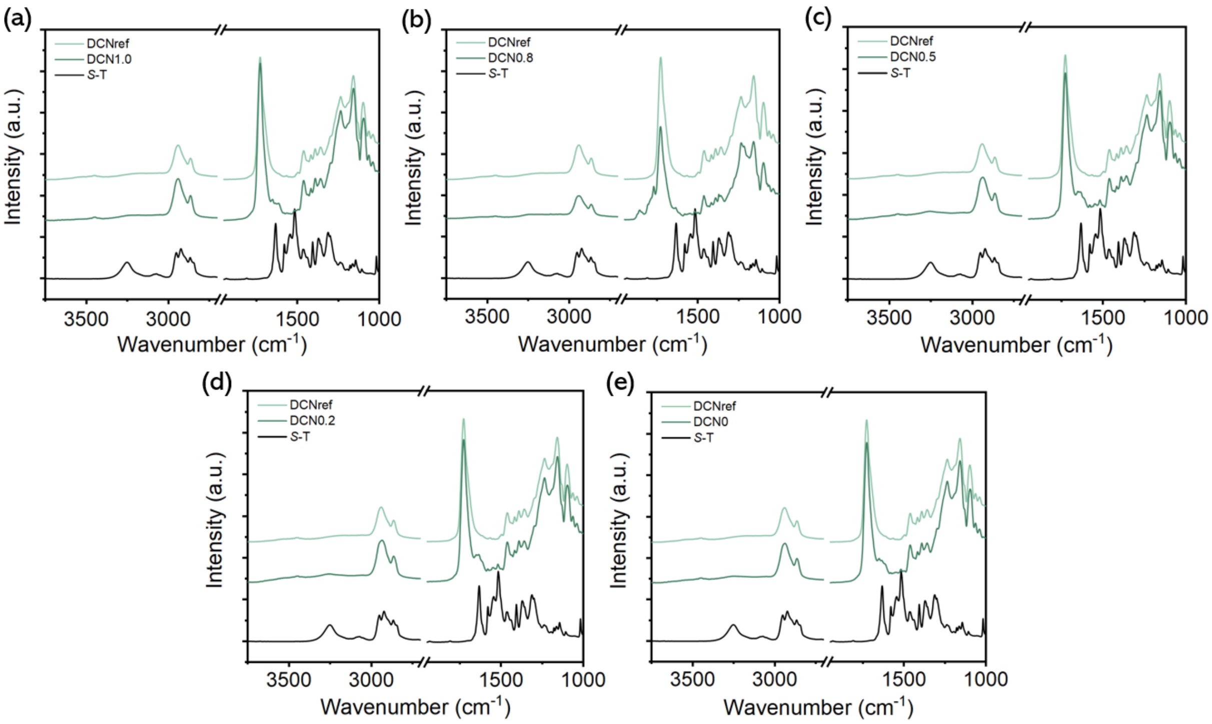

Figure S4. Comparison of FTIR spectra of **DCNref** and ***S*-T** with (a) **DCN1.0**, (b) **DCN0.8**, (c) **DCN0.5**, (d) **DCN0.2** and (e) **DCN0**.

3.3 Polarized Optical Microscope images of dynamic covalent networks.

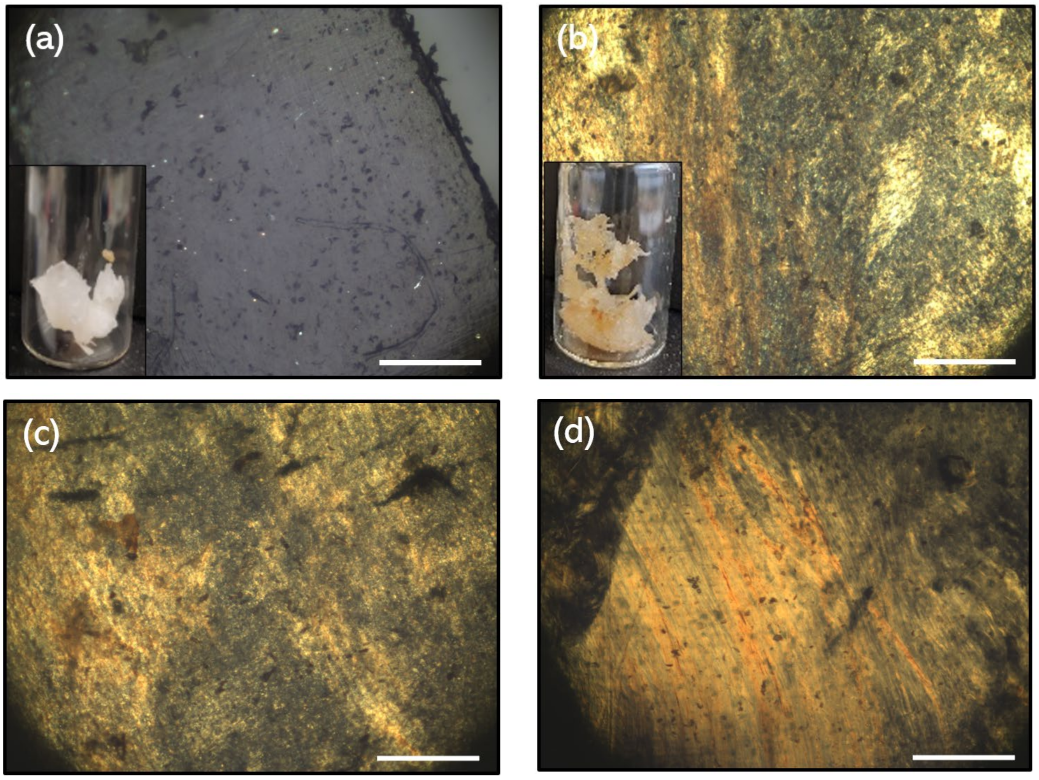

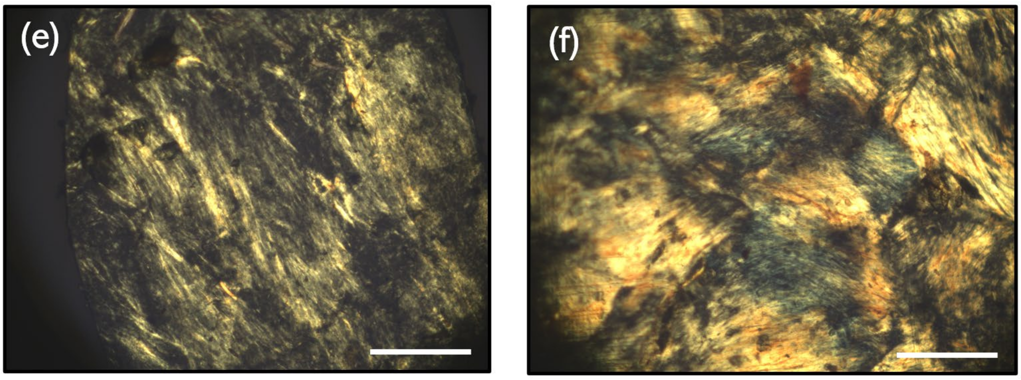

Figure S5. POM images of (a) **DCNref**, (b) **DCN1.0**, (c) **DCN0.8**, (d) **DCN0.5**, (e) **DCN0.2** and (f) **DCN0**. The inset scalebar represents 0.5 mm. The inset photographs in (a) and (b) show the vials containing unfilled and filled DCNs, respectively.

4. Mechanical properties of dynamic covalent networks

4.1 Tensile experiments.

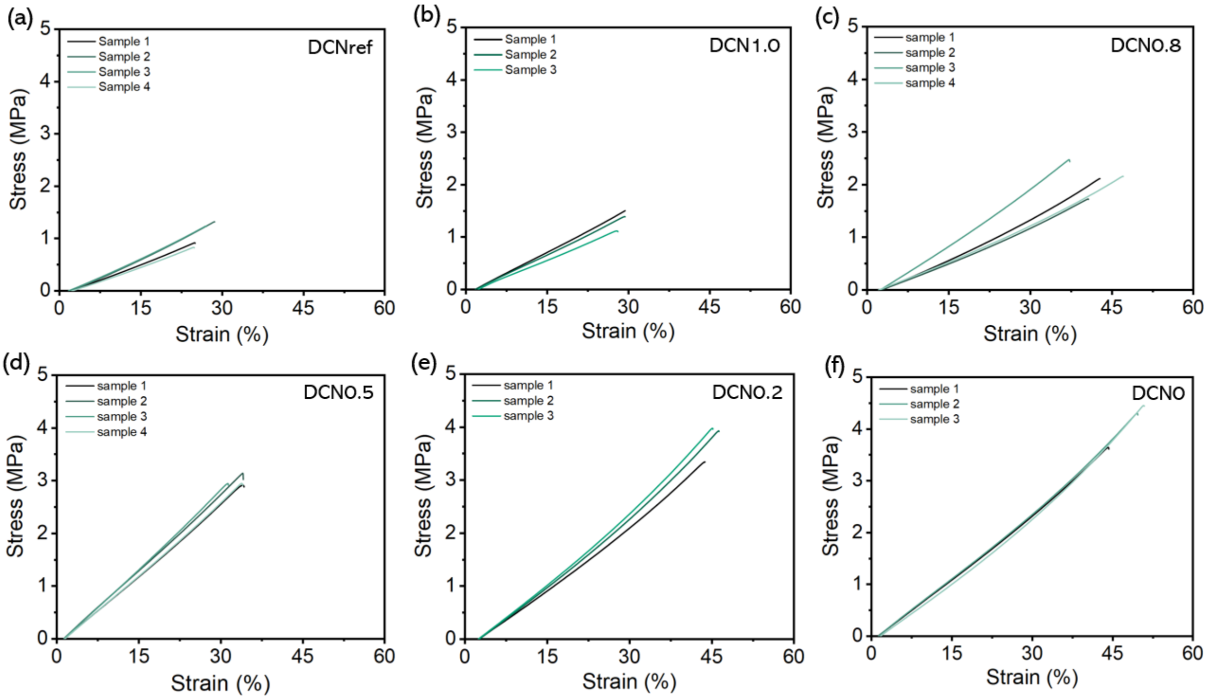

Figure S6. Stress-strain curves in tensile mode of (a) **DCNref**, (b) **DCN1.0**, (c) **DCN0.8**, (d) **DCN0.5**, (e) **DCN0.2** and (f) **DCN0**. A constant strain rate of 5 mm/min and a preload of 0.1 N were used for all experiments.

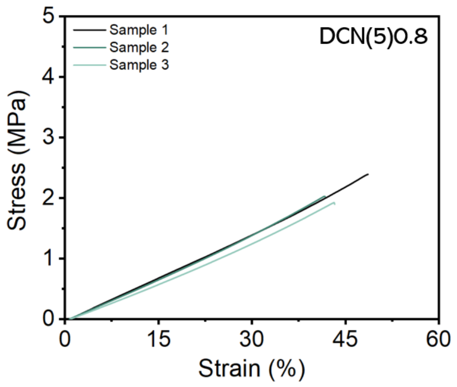

Figure S7. Stress-strain curves in tensile mode of **DCN(5)0.8**. A constant strain rate of 5 mm/min and a preload of 0.1 N were used for all experiments.

4.2 Single-edge notch experiments in tensile mode.

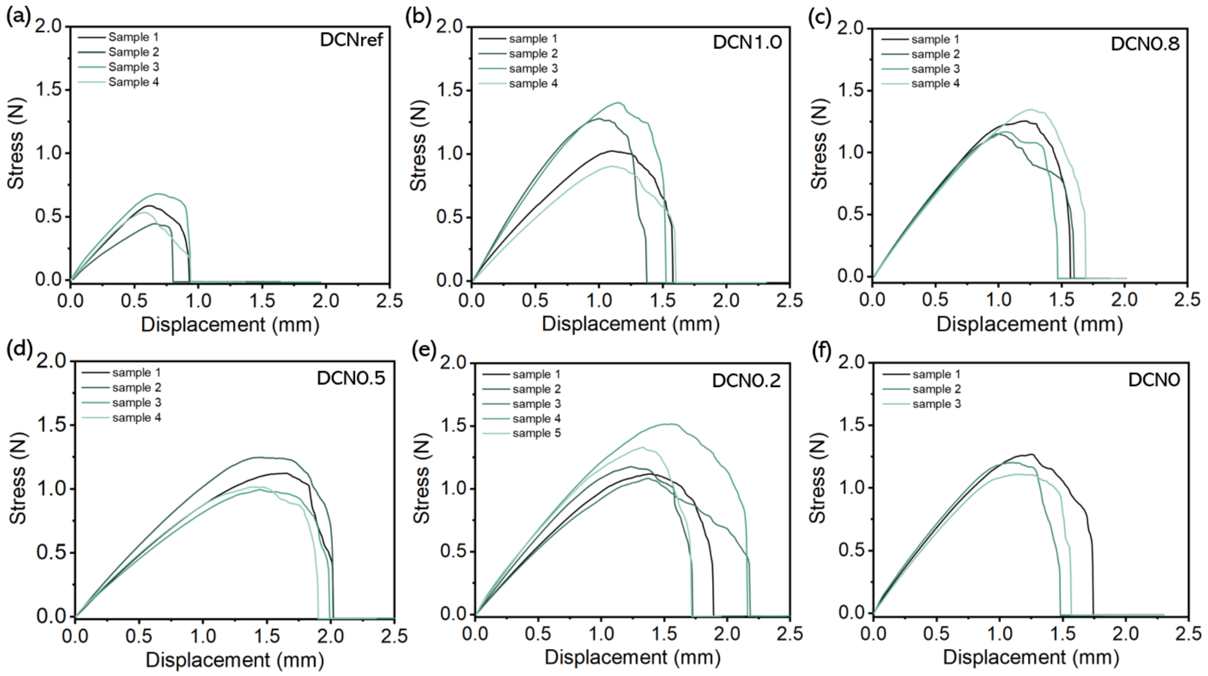

Figure S8. Force-distance curves by means of single-edge notch experiments in tensile mode on (a) **DCNref**, (b) **DCN1.0**, (c) **DCN0.8**, (d) **DCN0.5**, (e) **DCN0.2** and (f) **DCN0**. A constant strain rate of 5 mm/min was used for all experiments.

5. Stress relaxation experiments

Prior to the stress relaxation measurements, a thermal pre-treatment was applied to the samples in order to assure inconsistencies regarding different degrees of curing or sample roughness were eliminated. A stable signal was obtained within 0.1 s after the start of the measurement as shown by the representative stress relaxation measurement in Figure S9a. This observation prompted us to use the relaxation moduli at t = 0.1 s as the initial moduli G_0, which was in turn used to plot a normalized stress relaxation curve as shown in Figure S9b.

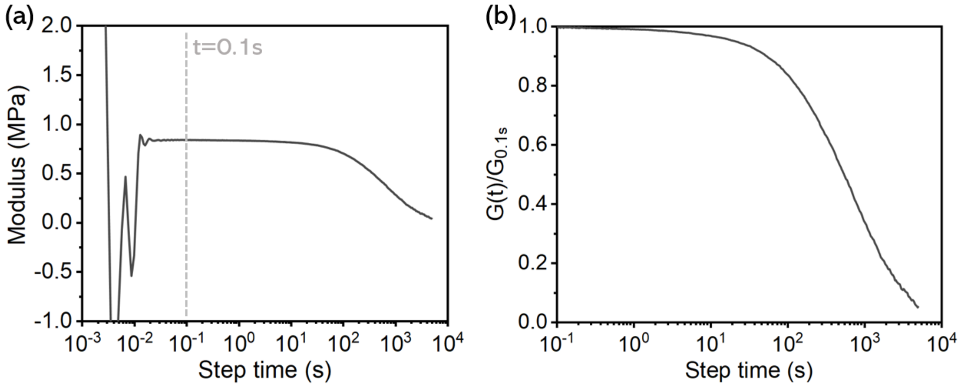

Figure S9. (a) A representative stress relaxation curve displaying the stabilization of $G(\mathrm{t})$ after 0.1 s. (b) The corresponding stress relaxation curve after normalization of $G(\mathrm{t})$ against $G(\mathrm{t} = 0.1\ \mathrm{s})$ (sample: **DCN0** at 160 °C).

In a next step, all stress relaxation curves were fitted using a stretched exponential model (Eq. S2).

Stretched exponential:

$$\frac{G(t)}{G_0} = e^{-(t/\tau*)^{\beta}} \qquad \text{(S2)}$$

With G(t) as the relaxation modulus at any point in time, G_0 as the relaxation moduli at t = 0, τ* as the characteristic relaxation time and β as the stretch exponential. The theoretically (stretch exponential model) determined τ* values as well as the stretch exponents for **DCNref**, **DCN1.0**, **DCN0.5** and **DCN0** are tabulated in Table S2. Furthermore, the moderate β values are indicative of a distribution of stress relaxation modes in the material, most likely due to inhomogeneous network formation.

Table S2. The theoretically (stretch exponential model) determined τ* and β values at 180 °C for **DCNref**, **DCN1.0**, **DCN0.5** and **DCN0**.

DCN[a]	Stretched exponential	
	τ* [s][b]	β[c]
**DCNref**	$3.5 \cdot 10^3$	0.62
**DCN1.0**	$2.9 \cdot 10^3$	0.59
**DCN0.5**	$3.2 \cdot 10^3$	0.66
**DCN0**	$0.9 \cdot 10^3$	0.73

[a] Polymers as depicted in Figure 1.[b] τ* obtained from fitting a stretched exponential (Eq. S2) to the stress relaxation curve at 180 °C. [c] β obtained from fitting a stretched exponential (Eq. S2) to the stress relaxation curve at 180 °C.

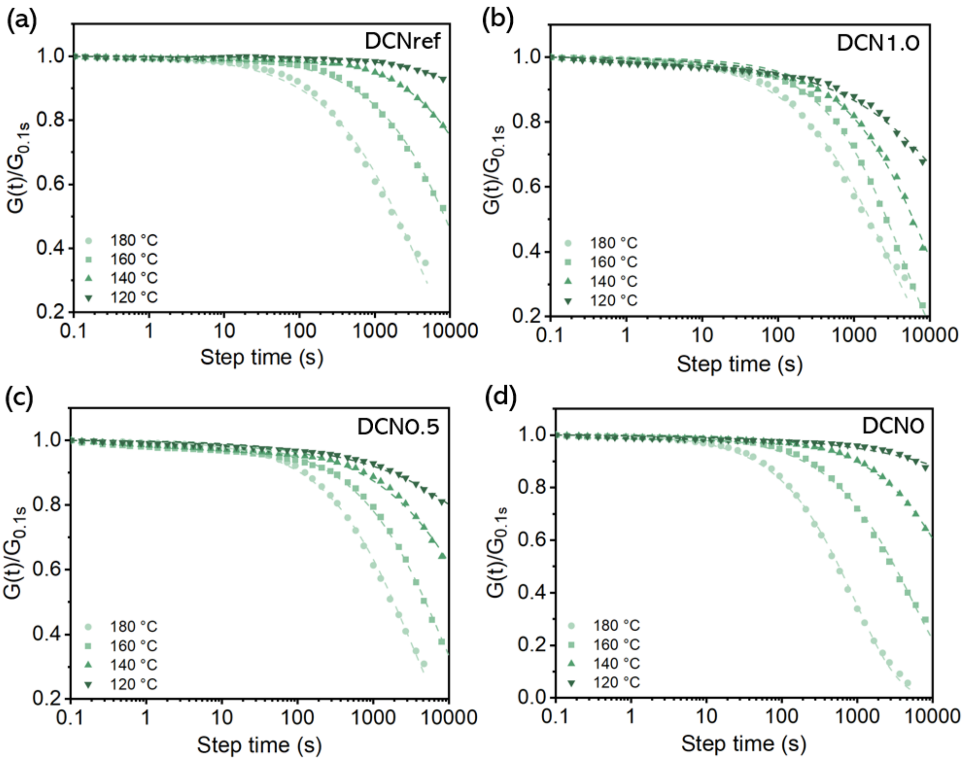

Figure S10. Oscillatory rheology stress relaxation experiments of (a) **DCNref**, (b) **DCN1.0**, (c) **DCN0.5**, (d) **DCN0** measured at 120, 140, 160 and 180 °C. The intersections of the dotted lines with the stress relaxation curves are indicative of $G(t)/G_0 = 1/e$ and $t = \tau^*$. A 1% step strain and an axial force of 0.5 N have been used for all experiments.

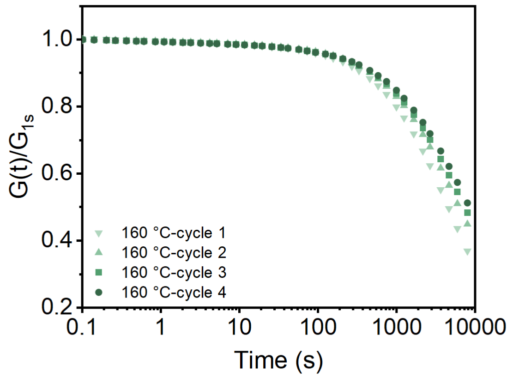

Figure S11. Oscillatory rheology stress relaxation experiments of **DCN0.5** measured at 160 °C over multiple cycles. The intersection of the dotted line with the stress relaxation curves are indicative of $G(t)/G_0 = 1/e$ and $t = \tau^*$. A 1% step strain and an axial force of 0.5 N have been used for all experiments.

6. Thermal properties of dynamic covalent networks

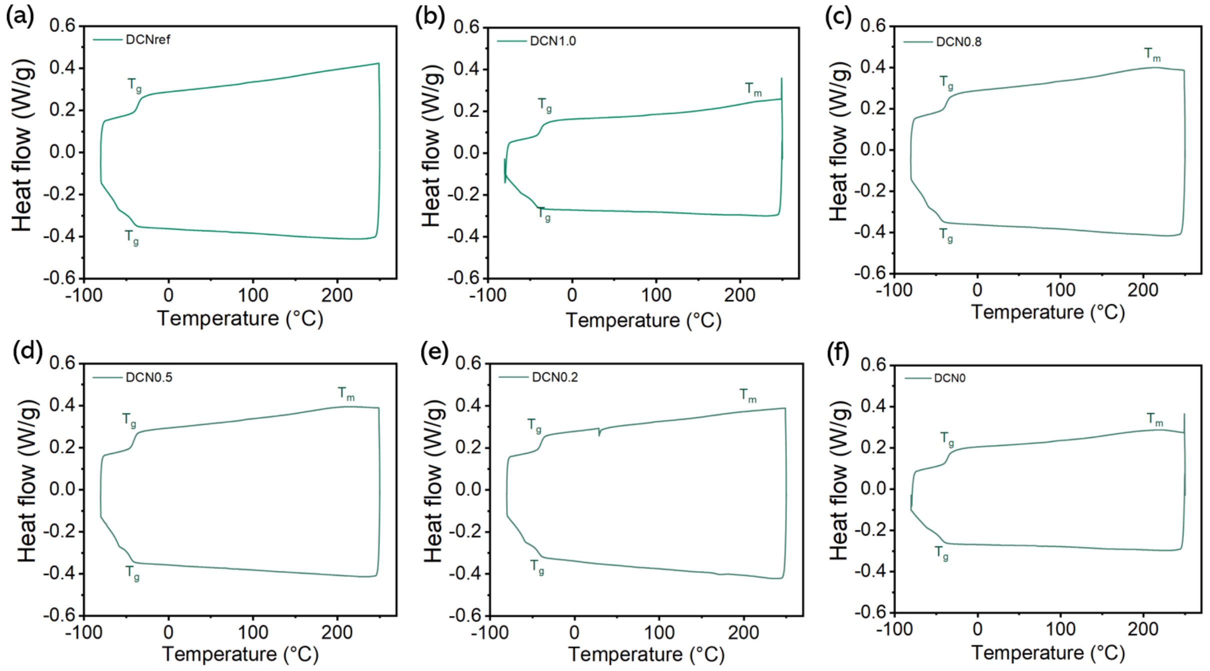

Figure S12. DSC traces of (a) **DCNref**, (b) **DCN1.0**, (c) **DCN0.8**, (d) **DCN0.5**, (e) **DCN0.2** and (f) **DCN0** (second heating and cooling run). Endothermic heat flows exhibit a positive value. A temperature ramp of 10 K min^{-1} was used for all experiments.

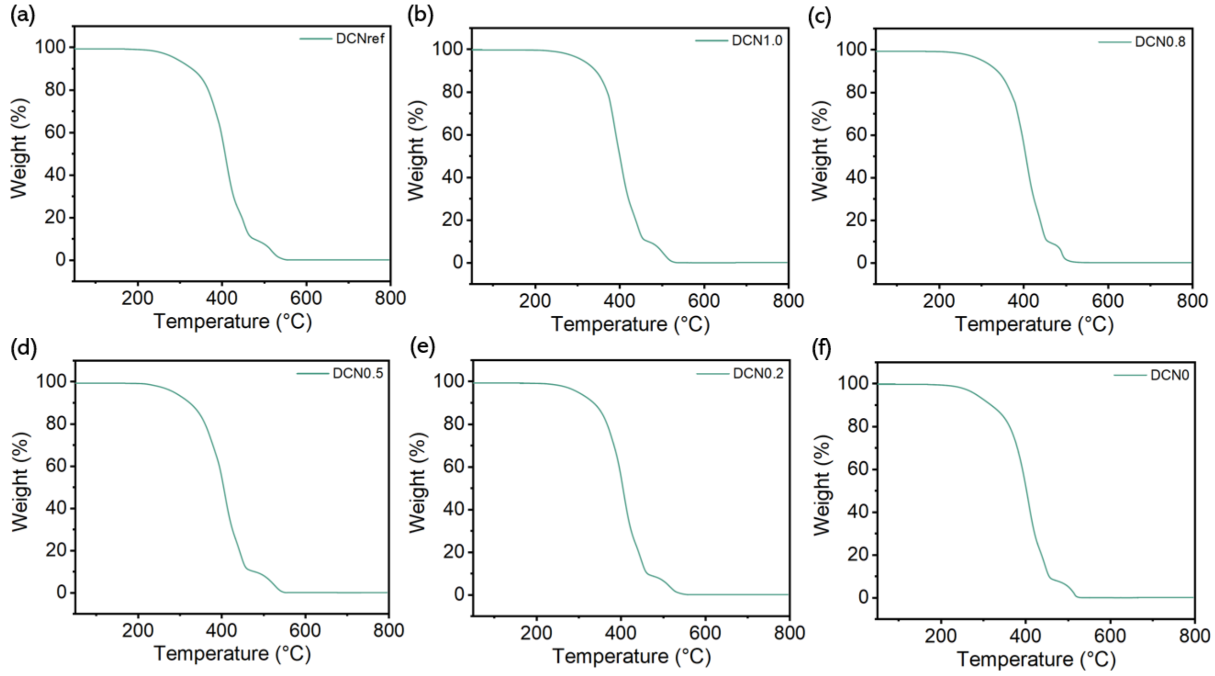

Figure S13. TGA traces of (a) **DCNref**, (b) **DCN1.0**, (c) **DCN0.8**, (d) **DCN0.5**, (e) **DCN0.2** and (f) **DCN0**. A temperature ramp of 20 K min^{-1} was used for all experiments.

7. Variable temperature infrared spectroscopy

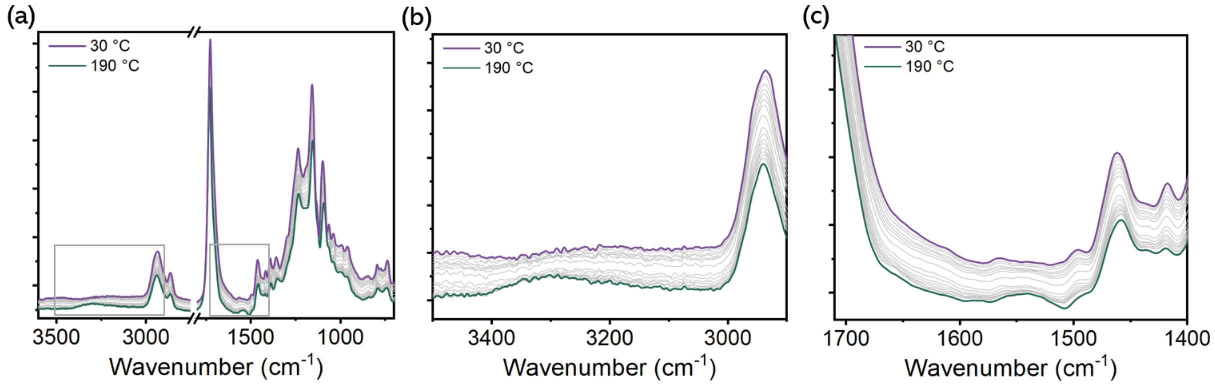

Figure S14. Variable temperature ATR spectra of (a) **DCNref** and corresponding zooms of the (b) N-$H_{stretching}$ region 3500 – 2900 cm^{-1} and (c) N-$H_{bending}$ region 1710 – 1400 cm^{-1} region. A temperature ramp of 5 K min^{-1} was used for all experiments.

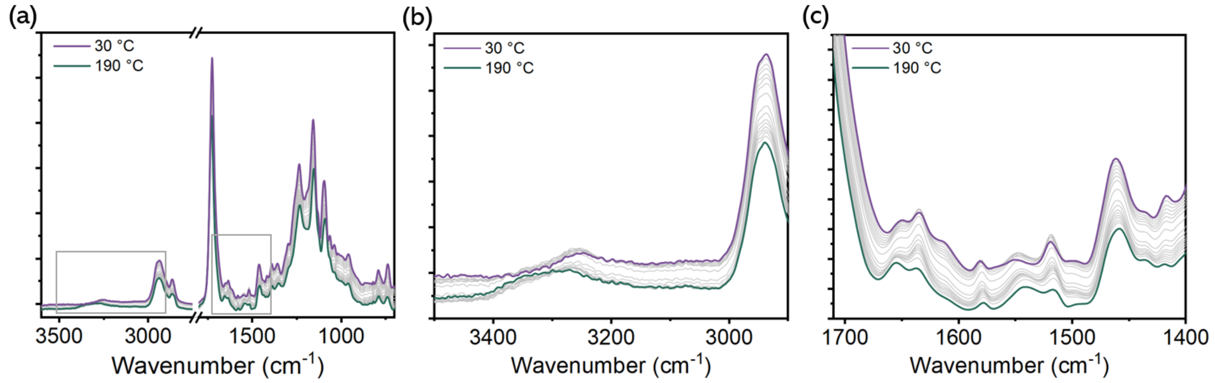

Figure S15. Variable temperature ATR spectra of (a) **DCN1.0** and corresponding zooms of the (b) N-$H_{stretching}$ region 3500 – 2900 cm^{-1} and (c) N-$H_{bending}$ region 1710 – 1400 cm^{-1} region. A temperature ramp of 5 K min^{-1} was used for all experiments.

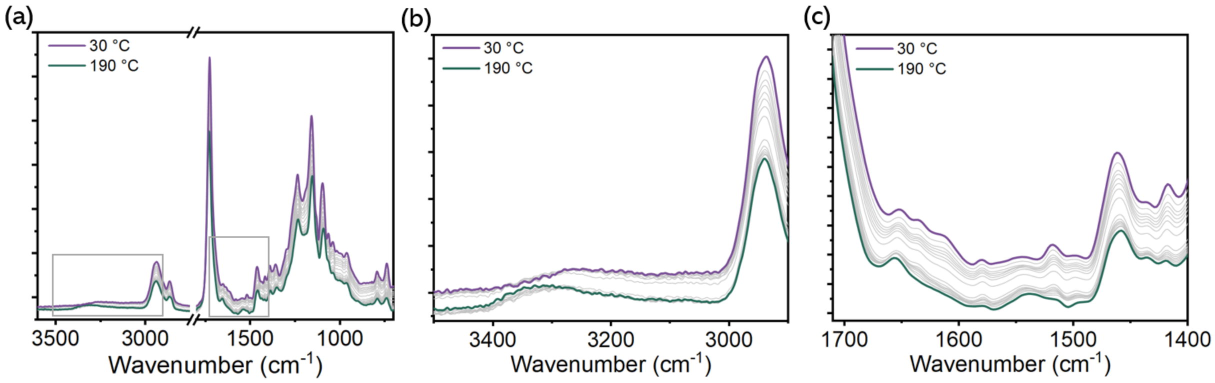

Figure S16. Variable temperature ATR spectra of (a) **DCN0.5** and corresponding zooms of the (b) N-$H_{stretching}$ region 3500 – 2900 cm^{-1} and (c) N-$H_{bending}$ region 1710 – 1400 cm^{-1} region. A temperature ramp of 5 K min^{-1} was used for all experiments.

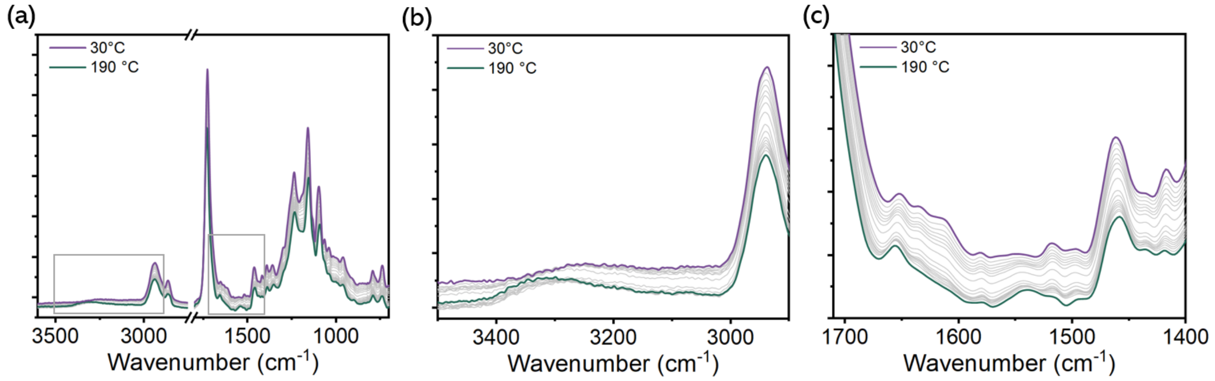

Figure S17. Variable temperature ATR spectra of (a) **DCN0** and corresponding zooms of the (b) N-$H_{stretching}$ region 3500 – 2900 cm^{-1} and (c) N-$H_{bending}$ region 1710 – 1400 cm^{-1} region. A temperature ramp of 5 K min^{-1} was used for all experiments.

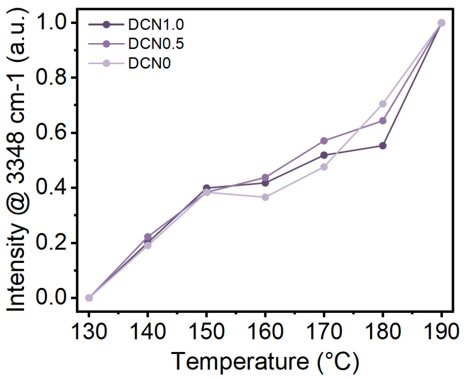

Figure S18. The normalized peak intensity at 3348 cm^{-1} as a function of temperature for **DCN1.0**, **DCN0.5** and **DCN0**. After the initial shift of the N-$H_{stretch}$ peak maximum (Figure S14b-S17b), an increase in absorbance at 3348 cm^{-1} is observed from 130 °C to 190 °C, which is generally indicative of non-hydrogen bonded N-H. This observation implies that ***S*-T** clusters are partially debonding and in turn start contributing to the material's dynamics.

8. Recycling & recovery experiments

8.1 Recycling and reinforcement recovery procedure.

For the recycling experiment, all samples were cut into small pieces and remoulded at 150 °C and 20 MPa for 2 hours (25 [length] x 10 [width] x 0.7 [thickness] mm). Sequentially, the reshaped samples were slowly cooled to room temperature before removal followed by cutting them into the desired dogbone shape (25 [length] x 2 [width] x 0.7 [thickness] mm). After each reprocessing cycle (3), a tensile experiment was performed. Prior to each measurement, the samples were dried at 120 °C at 21 mbar for 16 hours in a vacuum oven.

In order to demonstrate the ability to recover the reinforcement material, we submerged a part of the compression moulded dogbone samples (0.2 g) into 2 mL of methanol and 100 μL of triethylamine, which functions as a catalyst. After heating the solution to 60 °C for 8 hours, the cloudy suspension was allowed to cool to room temperature for 1 h. Subsequently, the suspension was centrifuged at 2500 rpm for 5 minutes followed by decanting the liquid into a separate vial. After

repeating the chemical redissolution procedure for 4 hours, the remaining solids were separated from the liquid, washed with methanol once and vacuum dried at 60 °C for 24 h, yielding 63% ***S*-T** exceeding 95% purity (Figure S20).

*8.2 Tensile experiments on recycled **DCNref** and **DCN0.5**.*

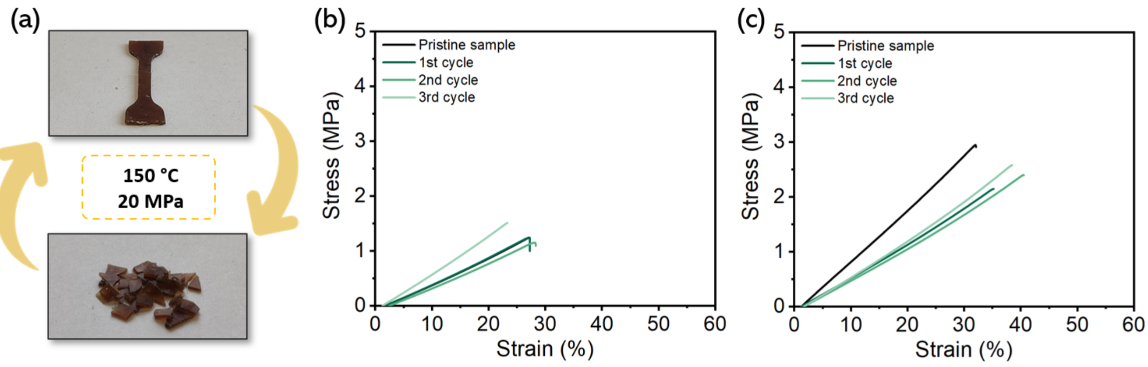

Figure S19. (a) Photographs demonstrating the reprocessability of the DCNs via compression moulding. Stress-strain curves of (b) **DCNref** and (c) **DCN0.5** before and after the 1st, 2nd and 3rd cutting and compression moulding cycle. A constant strain rate of 5 mm/min was used for all experiments.

*8.3 ^{1}H NMR spectra of **S-T** upon chemical recycling of **DCN0.5**.*

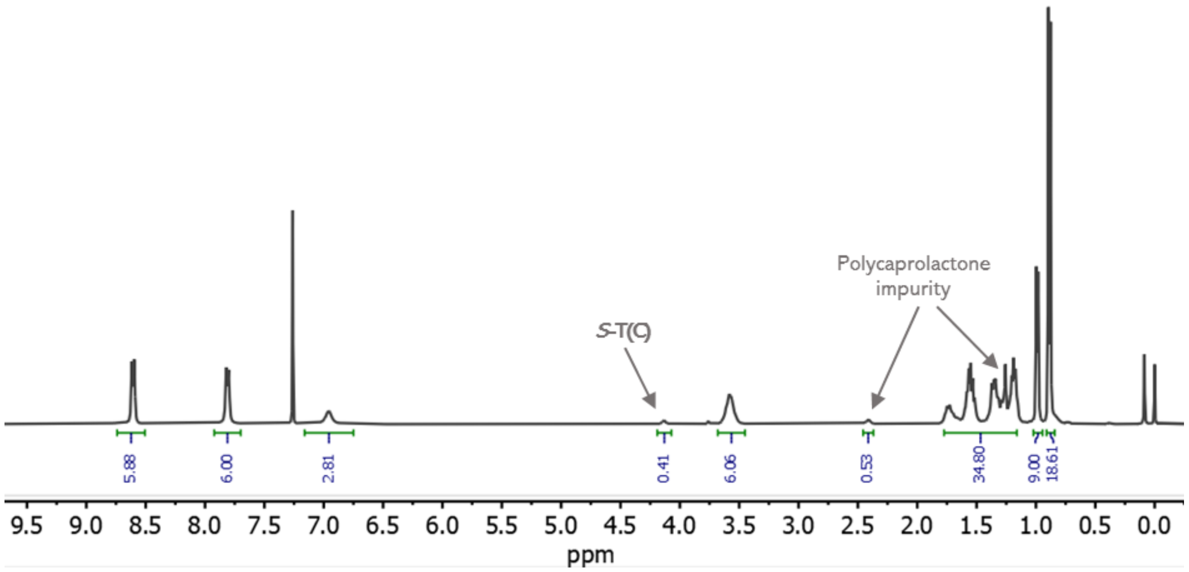

Figure S20. ^{1}H NMR spectrum of isolated ***S*-T** molecules in $CDCl_3$/TFA_{d1} (400 MHz, 25 °C) after chemical recycling. A small fraction of impurity remains around 2.41 and 1.26 ppm originating from the polycaprolactone fraction of the DCN matrix.

9. References

[1] H. Su, S. A. H. Jansen, T. Schnitzer, E. Weyandt, T. R. Andreas, J. Liu, G. Vantomme, E. W. Meijer, *J. Am. Chem. Soc* **2021**, *143*, 17128–17135.

[2] H. Zhang, S. Majumdar, R. A. T. M. Van Benthem, R. P. Sijbesma, J. P. A. Heuts, *ACS Macro Lett.* **2020**, *9*, 272–277.
